# Application of Near-Infrared Fluorescence With Indocyanine Green in Head and Neck Surgery: Advantages and Unexpected Findings From an Initial Experience in Mexico City

**DOI:** 10.7759/cureus.93725

**Published:** 2025-10-02

**Authors:** Ariel Martinez-Onate, Alan de Jesús Martínez Salas

**Affiliations:** 1 Surgery, Hospital Ángeles del Pedregal, Mexico City, MEX; 2 Urology, Hospital General de Tláhuac "Dra. Matilde Petra Montoya Lafragua", México City, MEX; 3 Urology, Hospital Ángeles del Pedregal, Mexico City, MEX

**Keywords:** fluorescence angiography, fluorescence guided surgery, fluorescence lymphography, head and neck surgery, thyroid and parathyroid surgery

## Abstract

Background: Fluorescence-guided surgery (FGS) using indocyanine green (ICG) for fluorescent angiography and lymphography is well established as a safe and reproducible technique in head and neck surgery, mostly for thyroid and parathyroid surgery.

Methods: We present our experience with FGS for thyroid and parathyroid gland (PG), and other complex surgeries in the head and neck region, such as major gland resections, lymph node dissections, and carotid body tumor resections, among others.

Results: A total of 33 patients underwent FGS using ICG in different doses. A total of 50 PGs were identified; two carotid paragangliomas, two major salivary glands, the thoracic duct, and its right side equivalent were observed emitting fluorescence through the supraclavicular hollow. Twenty-one nerves were also observed emitting fluorescence.

Conclusions: FGS proved to be an excellent tool for additional surgical-related benefits and surgical safety in head and neck surgery, especially in complex procedures.

## Introduction

Fluorescence-guided surgery (FGS) using indocyanine green (ICG) as a dye takes advantage of the fluorescence emitted by the ICG as it circulates in the arterial and venous system when it is excited by near-infrared (NIR) light of the proper wavelength after ICG is injected intravenously (IV). This is known as fluorescence angiography. ICG can also be injected subcutaneously or into the lymph nodes, and soon it is transported inside the lymphatic vessels, and this is known as fluorescence lymphography. Fluorescence angiography has been used in multiple laparoscopic procedures, such as colorectal and esophageal surgeries. ICG pharmacodynamics have already been studied. The liver extracts around 70% of the circulating ICG and eliminates it through the bile. This property is applied for the surgery of the gallbladder, biliary tree, liver, and pancreas [1,2]. Due to the fact that ICG contains a minimal amount of iodine, 125 mcg/mL of prepared ICG (USP) to improve its solubility, it is not recommended for patients with a previous allergy to iodine. Otherwise, its safety is widely known and is better than that of methylene blue in this regard. There are some tissues that capture ICG in a very early and intense manner, like the parathyroid glands (PGs).

Autofluorescence is a physical property of some tissues in our body when they are exposed to NIR light of a specific wavelength, and it is detected by a camera with optical biosensors without the need for an external fluorescent dye. PGs exhibit autofluorescence, which is used to detect PGs during surgery; however, autofluorescence does not give any information about the vascularity and viability of the glands. Ischemia of the PGs is considered a major risk factor for the development of postoperative surgical hypoparathyroidism (PoSH) [3].

We present our initial experience with ICG fluorescence angiography (ICG-FA) of the PGs and other structures in the head and neck. We started using ICG-FA for the surgery of the thyroid and parathyroid glands in January 2019. We underline the convenience of using surgical loupes with magnification 2.5X for the visual identification (V-Id) of the PGs, followed by confirmation with ICG-FA to evaluate the viability of the glands. We also took advantage of the safety and versatility of ICG-FGS in other surgical procedures of the head and neck; namely, major salivary gland resections, carotid paragangliomas (carotid body tumors) resection, and fluorescence lymphography for the visualization of the thoracic duct and its equivalent on the right side through the neck in the supraclavicular hollow. The purpose of this prospective study is to present our findings with the use of ICG-FGS in the head and neck region. 

## Materials and methods

This was a retrospective case series including cases from five different private hospitals (Hospital Ángeles del Pedregal, Hospital Ángeles Metropolitano, Hospital Santa Coleta, Hospital Servicura, and Hospital San Ángel Inn Sur) in Mexico City from January 2019 to October 2024, in which fluorescent angiography and lymphography with ICG were used during thyroid and parathyroid surgeries for benign and malignant pathologies and other surgical procedures in the head and neck area.

Hospital Angeles del Pedregal Research Board issued approval dated September 1, 2025. As the study is a retrospective case series, it is exempt from formal approval from the Ethics Committee or HAP IRB. This aligns with current national regulations, specifically NOM-012-SSA3-2012. Informed consent was signed by the patients or their legal guardians, and permission was granted for the recording and publication of patients' medical data and images, while ensuring anonymity of patient identification.

Study population

The inclusion criteria were adult patients (18 years old and older), of both sexes, undergoing a head and neck procedure of the kind described above, where FGS with ICG was employed. A total of 33 consecutive adult patients undergoing FCG-ICG were included.

Procedure

**Figure 1:**
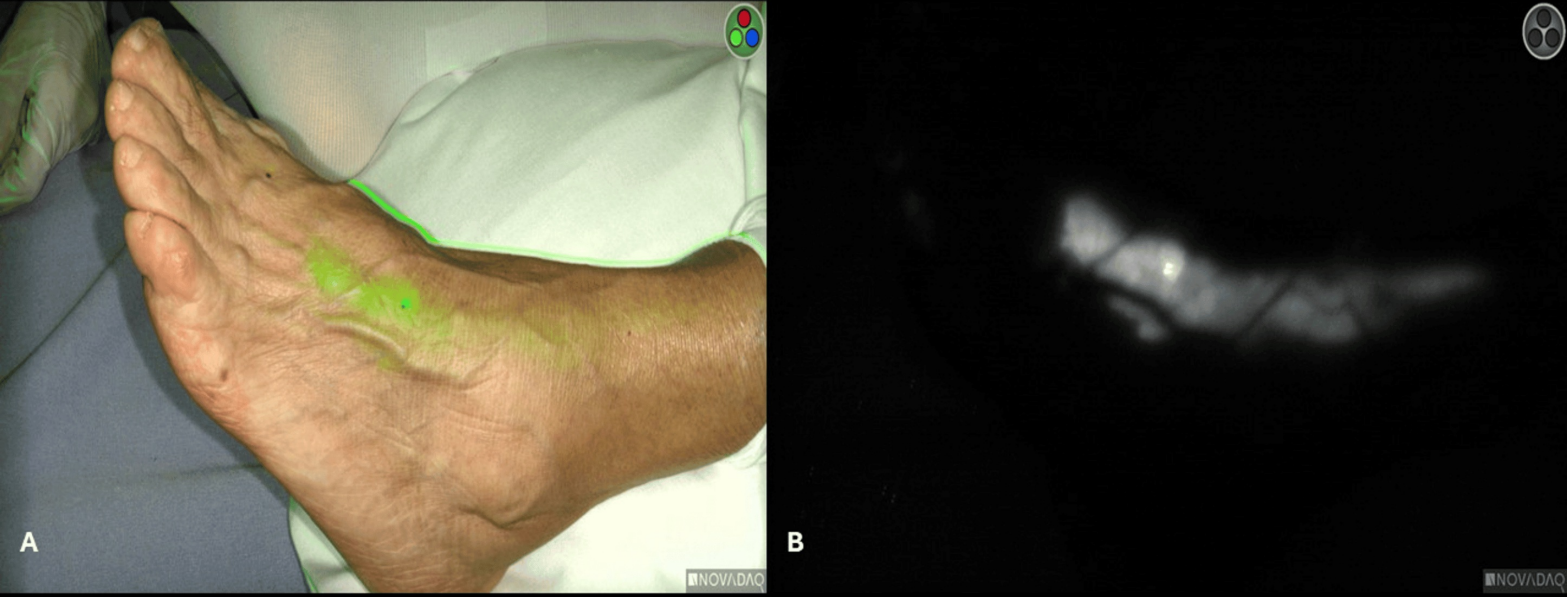
ICG in a lymphatic duct of of the dorsum of the foot. (A) Overlay mode. (B) Black and white mode ICG: indocyanine green

In all cases, the preparation of ICG employed was 25 mg in 10 mL of sterile water. For thyroid and parathyroid surgery, in our first 10 cases, we used a dose of 0.3 mg of ICG per kg of body weight. Afterwards, we used 2.5 mg IV in single doses, with an average of two shots per patient, depending on the extent of the surgery and the need to confirm the visualization and viability of parathyroid tissue [4]. In the cases of major salivary gland surgery, we applied 2 mL (5 mg) of ICG IV transoperatively in one or two doses. For fluorescence lymphography, we aimed to see the thoracic duct or its right-side equivalent through the neck in the supraclavicular hollow. In three patients, we alternatively injected 2 mg of ICG subcutaneously in the dorsum of one foot and 5 mg of ICG in the lymph nodes of each inguinal region guided by ultrasound (Figure 1).

Initially, we saw nerves emitting fluorescence as a finding during the revision of thyroid and parathyroid surgical videos. In these cases, the ICG dose that was employed was the same dose used to see the PGs. In three patients in whom we wanted to intentionally see the laryngeal nerves emitting fluorescence, a total of 3 mL (7.5 mg) was administered IV intraoperatively as a single dose in each case. We had two cases of carotid body tumor (CBT) for which two IV doses of ICG of 2.5 mg for each were administered to perform a fluorescent angiography of the tumors before their resection to delimit their borders, and for the confirmation of complete resection and permeability of the carotid bifurcation and of the internal and external carotid arteries after the lesions were resected.

Five different fluorescence open surgery cameras were employed during our study: IC-Flow™ (Diagnostic Green Ltd, Athlone, Ireland), Storz Image 1™ with a Vitom™ telescope (Karl Storz SE & Co. KG, Tuttlingen, Germany), Novadaq-Stryker 1688 AIM™ and Pinpoint™ (Stryker Corporation, Kalamazoo, Michigan, United States), and VisionSense™ (Medtronic plc, Galway, Ireland). In one patient, it was possible to compare the image of two different systems, IC-Flow™ camera vs. Storz Image 1™ with a Vitom™ telescope.

As the surgical procedures were performed in five different private hospitals, which had different equipment for clinical blood test analysis, this limited the possibility of assessment of postoperative serum intact parathyroid hormone (iPTH), calcium, phosphorus, and albumin in all cases. Thyroid and parathyroid surgery patients were clinically explored postoperatively on the day of the surgery and at 12 hours and 24 hours after surgery, looking for clinical signs of hypocalcemia, and were interrogated about any symptoms as well.

Thyroidectomies and neck explorations for parathyroid procedures were performed according to the standardized technique with either a transverse cervical or apron incision in the central inferior neck. Nerve monitoring of the laryngeal nerves was employed in all cases of thyroid and parathyroid resections, and facial nerve monitoring was employed in the major salivary gland surgeries as well. The thyroid gland was completely exposed, and we initially looked for the V-Id of the PGs with surgical loupes, magnification 2.5X. Meticulous subcapsular dissection of the PGs was performed, avoiding damage to the glands and their vascular supply [3].

In one case, we tried to identify the PGs with ICG-FA with the thyroid gland in situ, but the emission of fluorescence from the thyroid was so intense that it didn't allow us to see anything else. In the rest of the patients, V-Id was repeated after the surgical specimen of the thyroid gland, and the lymph node dissection specimen was extracted. After V-Id confirmation was done, the ICG was injected IV in the different doses that were employed according to the evolution of our protocol, and the fluorescent angiography confirmation of the parathyroid nature of the tissue was recorded in video and still photographs. The vascularity was confirmed visually with magnification and with ICG-AF.

**Figure 2:**
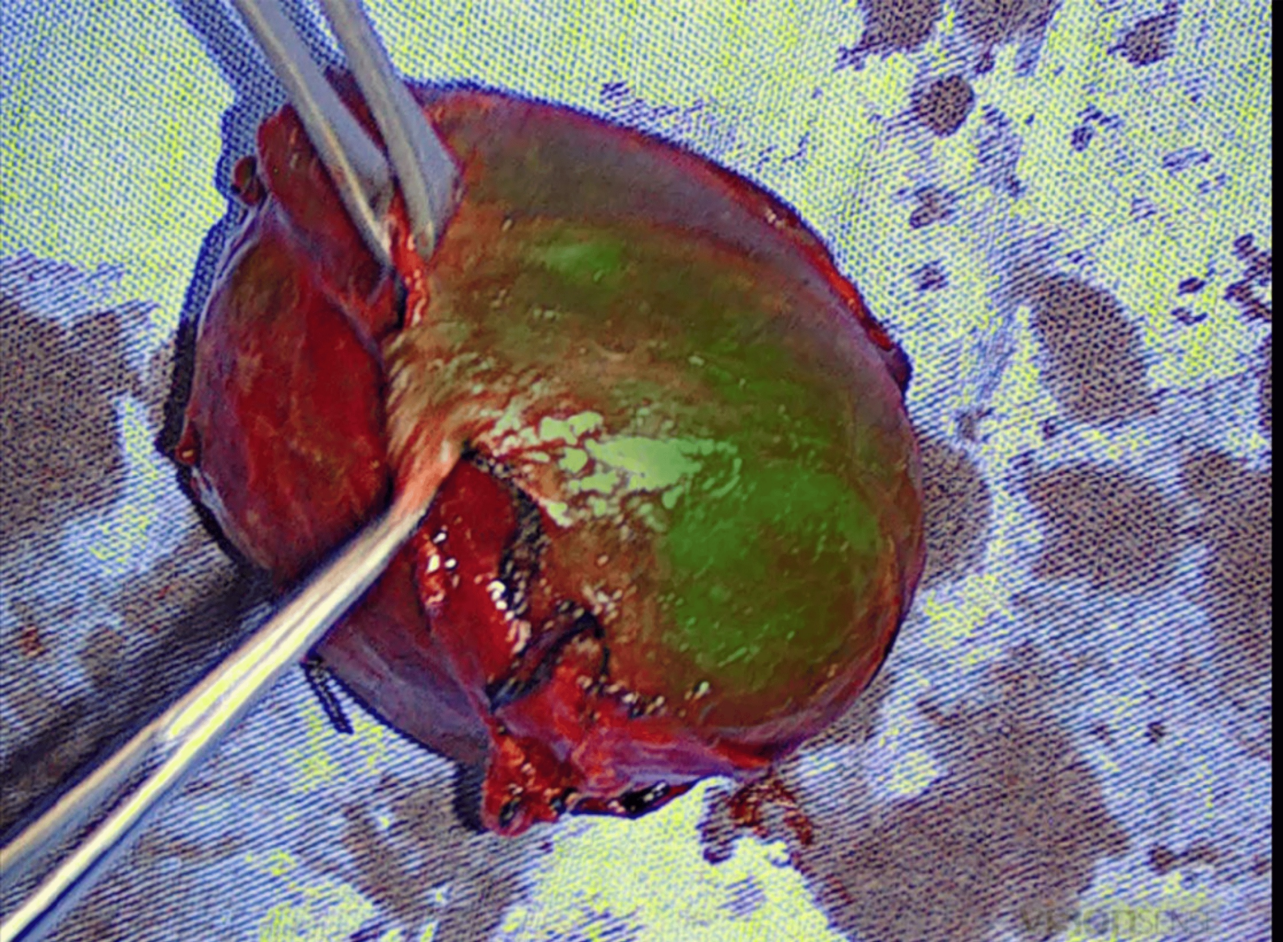
Revision of thyroidectomy specimen looking for resected parathyroid glands in overlay mode.

For the surgery of the PGs, fluorescent angiography was employed for confirmation when the dissected PGs were identified first by V-Id with magnification. For parathyroid hyperplasia, a subtotal parathyroidectomy was performed, and in the cases of parathyroid adenoma, a direct resection was done, preserving the recurrent laryngeal nerve; in these cases, fluorescent angiography was employed for the identification and vascular examination of the remaining PGs. For thyroidectomies, the camera was employed to examine the thyroid specimen once it was removed, looking for any accidentally resected PGs (Figure 2).

A superficial lobectomy of the right parotid gland was performed for a pleomorphic adenoma in coincidence with a left hemithyroidectomy. A preauricular incision extended to the posterior part of the angle of the jaw was employed. Nerve monitoring of the branches of the right facial nerve was employed, and all the branches of the facial nerve were respected. For the resection of a submandibular gland on the right side, an incision approximately three finger breadths below the inferior border of the mandible was employed, and nerve monitoring of the inferior branches of the facial nerve was employed. In the two cases of carotid paraganglioma, an oblique incision of approximately 6-7 cm was done, and cranial nerves X and XII were identified and referred. Proximal reference of the carotid artery was carried out, and an adequate dissection and separation of the deep jugular vein was performed. Subadventitial dissection of the tumor was done employing a Ligasure Exact Dissector™ (Medtronic plc) instrument for hemostasis and dissection. For major lymphatic ducts visualization, the thoracic duct in the left supraclavicular hollow, and its equivalent on the right side, were searched after the lymph node dissection and/or extraction of surgical specimens.

The visualization of the nerves that emitted fluorescence was concomitant with the original thyroid and parathyroid surgeries. The nerves were the recurrent laryngeal and the external branch of the superior laryngeal nerve bilaterally. Confirmation of the neural nature of the structures emitting fluorescence was done with the nerve monitoring system. In the case of the brachial plexus and phrenic nerve on the right side, the size of those nerves and their anatomical position were obvious, and so, these were identified visually. Videos and still photographs of all the procedures were reviewed postoperatively in search of nerves that emitted fluorescence with the ICG. The nerves that depicted this feature are described in the results.

## Results

A total of 33 patients were included in this study, 27 women and six men. Ages ranged from 16 to 83 years, with an average of 51.3 years. Fifty PGs were evaluated in 27 patients. A total of 20 thyroid surgeries were done, which included nine lobectomies, 10 total thyroidectomies, one completion thyroidectomy, and two resections of lymph nodes for metastases of a papillary thyroid cancer after incomplete neck dissections. The indications for the thyroid procedures were papillary thyroid cancer in 11 cases, and follicular adenomas and goiter in nine cases. Two subtotal parathyroidectomies for parathyroid hyperplasia and five resections for single parathyroid adenoma were performed.

**Figure 3:**
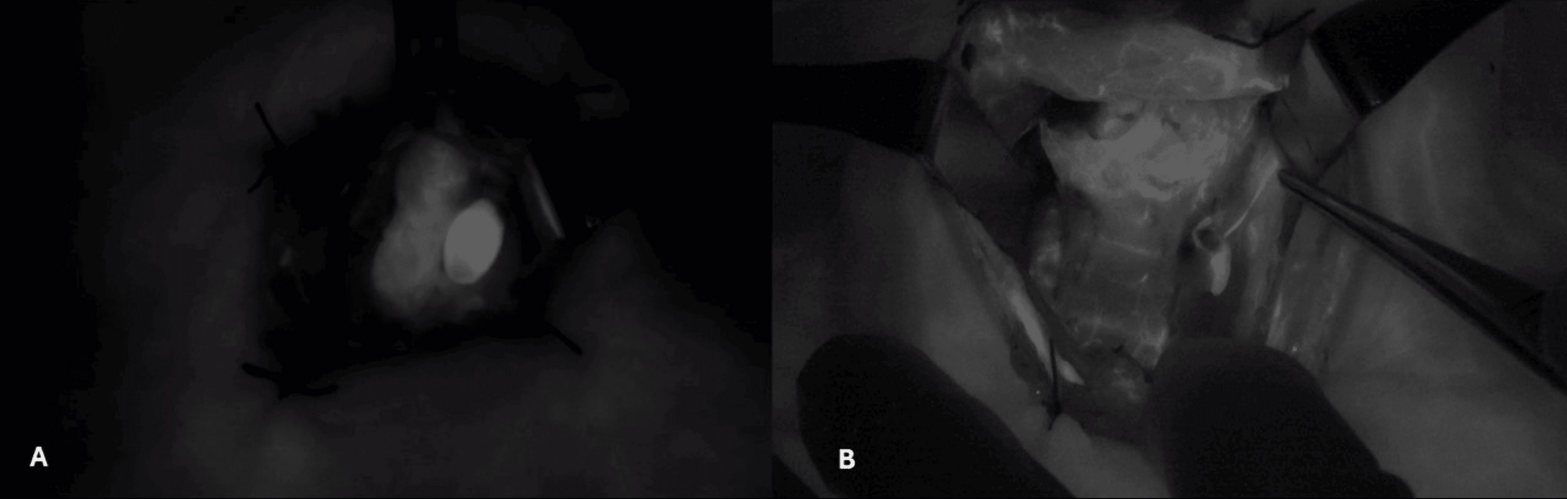
(A) Left parathyroid adenoma. (B) Left upper parathyroid gland with its arterial pedicle. Both are in black and white mode.

The ICG Score of the PGs was determined according to the previously described score by Fortuny et al. [3]. In an ICG Score 0, the PG is dark black after the injection of ICG, which indicates that the gland is not vascularized. In an ICG Score 1, the PG is grey or heterogeneous, indicating that the gland is partially vascularized. In an ICG Score 2, the PG is white, it indicates that the gland is well-vascularized (Figure 3). One PG (2%) displayed an ICG score of 0, seven (14%) displayed an ICG score of 1, and 42 (84%) displayed an ICG score of 2.

In the case in which the two different camera systems, IC-Flow™ and Storz Image 1 Vitom™, were compared, the images obtained with the first system were clearer and brighter in the monochromatic mode. In two patients of our series, we considered that fluorescent angiography of the PGs was not useful. Both had had previous extensive neck surgery for papillary thyroid cancer, and we deduced that the resultant fibrosis prevented us from visualizing the PGs with fluorescence.

One patient with papillary thyroid carcinoma and hyperparathyroidism developed PoSH clinically and biochemically. Total thyroidectomy and resection of the right lower inferior parathyroid adenoma were performed. The remaining three PGs were given an ICG Score of 1; the vascular twigs of the remaining PGs were visible with fluorescence, but the PGs remained gray. Albumin-corrected calcium reached a low level of 7.8, 48 hours after surgery. The patient received vitamin D and 1.5 g of Tums with a complete resolution of PoSH after three months.

In another patient who underwent a total thyroidectomy and a bilateral central lymph node dissection, four PGs were observed once the surgical specimen was removed. An ICG Score of 1 was given to the PGs, but the patient didn´t develop any clinical or biochemical data of PoSH. Since we started using fluorescent angiography for the PGs either for surgery of the thyroid gland or for parathyroid pathology, we haven't had a case of permanent PoSH.

Two resections of lymph nodes for metastases of papillary thyroid cancer after total thyroidectomy and incomplete neck dissections were performed. No parathyroid glands were observed during either surgery. Two procedures on the major salivary glands were performed. One superficial parotidectomy for pleomorphic adenoma in a patient who underwent a left hemithyroidectomy as well. One submandibular gland resection for suspicion of metastases from a papillary thyroid carcinoma based on the results of an I-131 scan. Two CBTs, 1 Shamblin II, and 1 Shamblin II-III were operated. One of the CBTs was resected in a patient who underwent a left hemithyroidectomy as well. 

There were some combined procedures in which FGS with ICG was employed for combined surgeries: (i) pne left thyroid lobectomy with resection of the superficial lobe of the right parotid gland with a pleomorphic adenoma, (ii) one total thyroidectomy for papillary cancer with resection of a right inferior parathyroid adenoma, and (iii) one left thyroid lobectomy with resection of a right carotid body tumor (CBT) Shamblin II-III.

**Figure 4:**
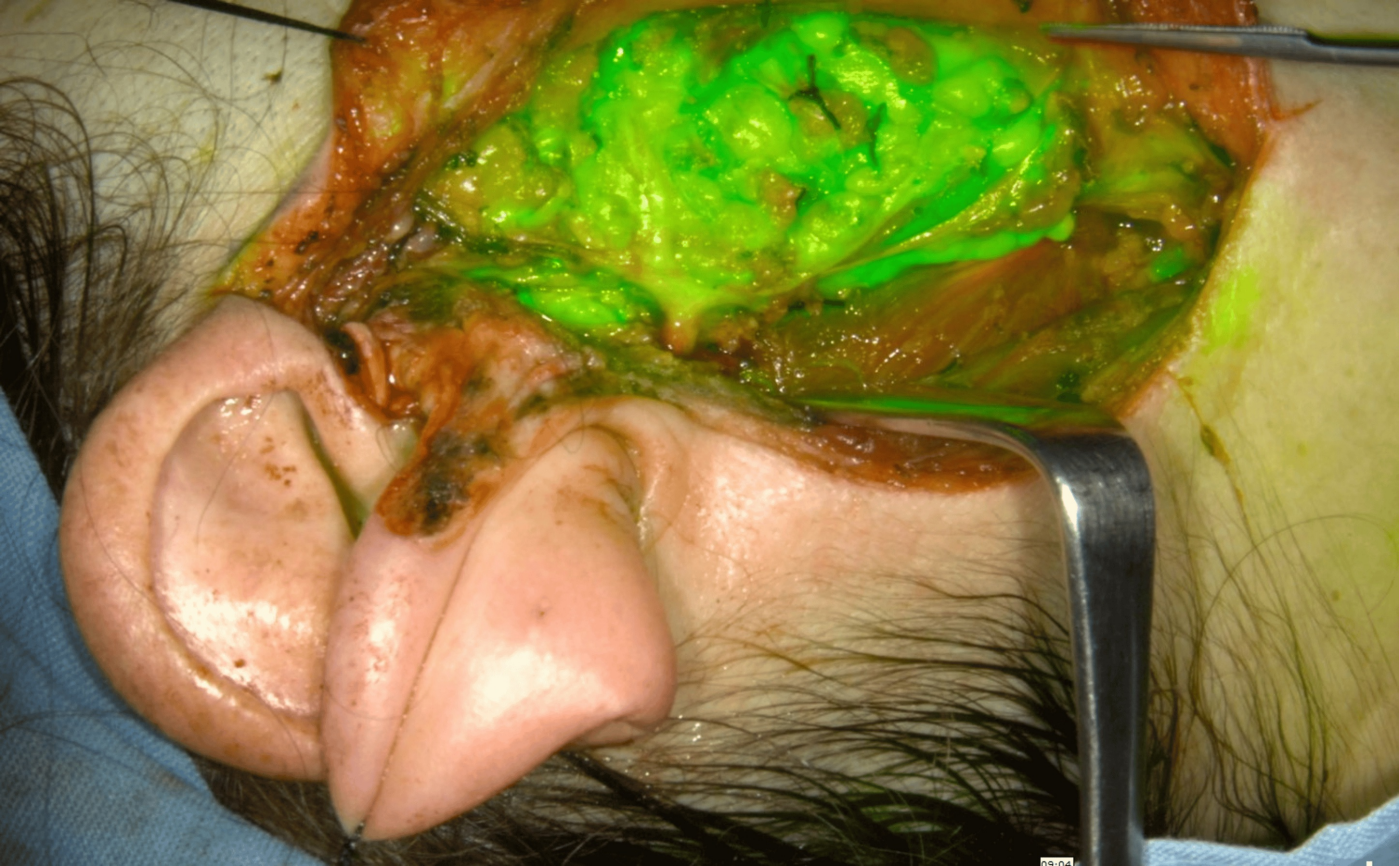
The deep lobe of the right parotid gland and the right facial nerve after superficial parotidectomy for a pleomorphic adenoma in overlay mode.

We discovered that major salivary glands rapidly emitted fluorescence (between 30 and 60 seconds after IV injection of 2 mL of ICG and in an intense manner; this was an unexpected finding during combined surgical procedures. We registered this finding in video and still images (Figure 4).

**Figure 5:**
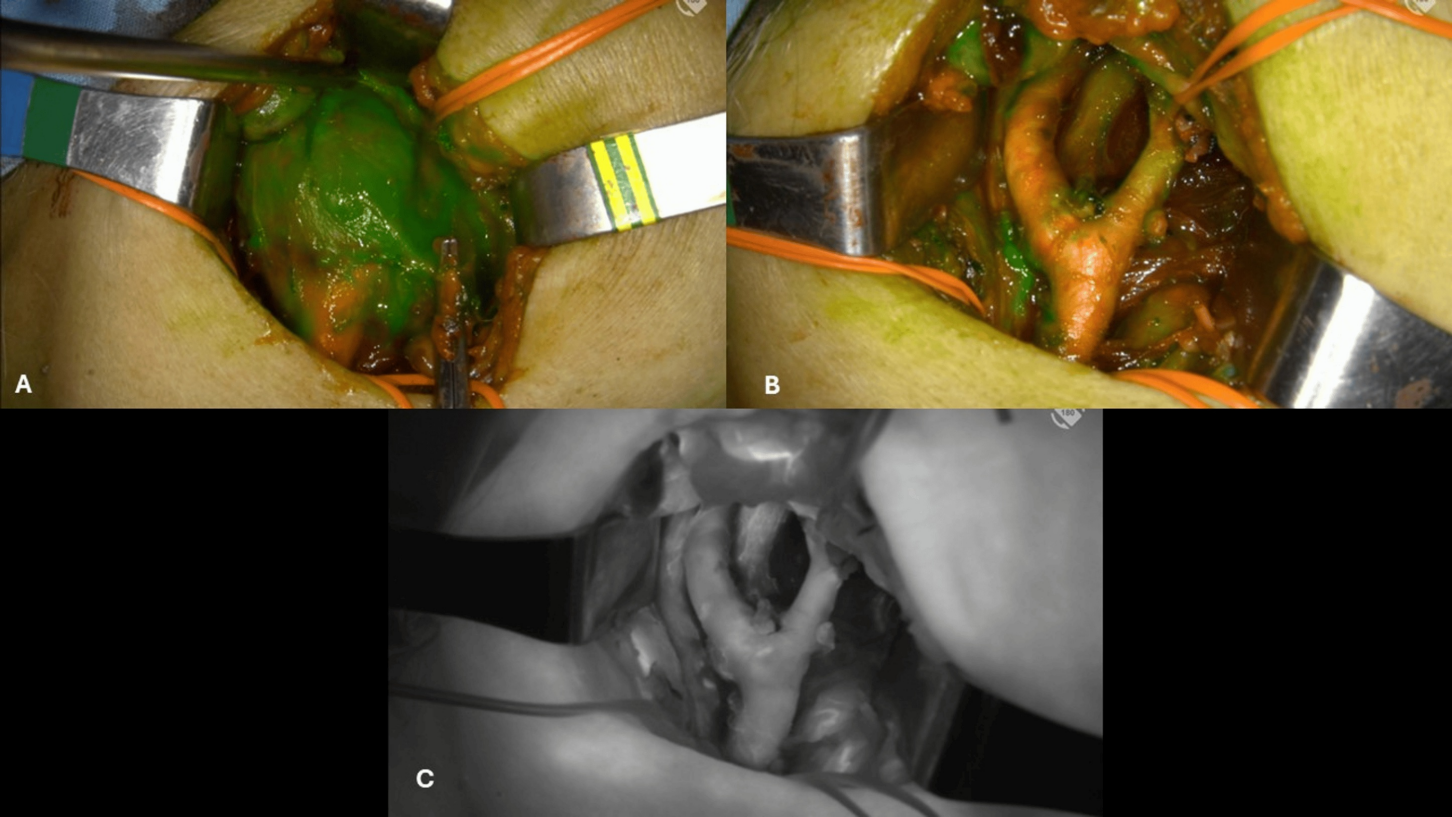
Right carotid paraganglioma. (A) Before resection, (B) Post-resection, both in overlay mode, (C) Carotid artery FGS arteriography post-resection in black and white mode. FGS: fluorescence-guided surgery

For the two cases of CBT, two doses of ICG of 2.5 mg were given per patient. The first dose was administered to perform fluorescent angiography of the tumors before resection to delimit their borders. After the lesions were resected, a second dose of 2.5 mg was injected for the confirmation of a complete resection and permeability of the carotid bifurcation and of the internal and external carotid arteries after the manipulation of the carotid bifurcation (Figure 5). In one patient with infiltration of the right-sided carotid sheath by metastatic tissue of a PTC, the resection of most of the metastatic tissue was attempted. This implicated major manipulation of the common carotid artery on the right side. At the end of the partial resection of the metastatic tissue, fluorescent angiography of the right side common carotid artery was performed with the IV injection of 2 mg of ICG, which confirmed adequate patency of the carotid artery.

Fluorescence lymphography of the TD or its equivalent on the right side was attempted in three cases. ICG was injected in the dorsum of the foot and in the lymphatic nodes of the inguinal region with the assistance of ultrasound. The thoracic duct and its equivalent on the right side were visualized during the extraction of a thyroid nodule off the mediastinum on the left side and during a Group IV lymph node dissection on the right side, respectively.

**Figure 6:**
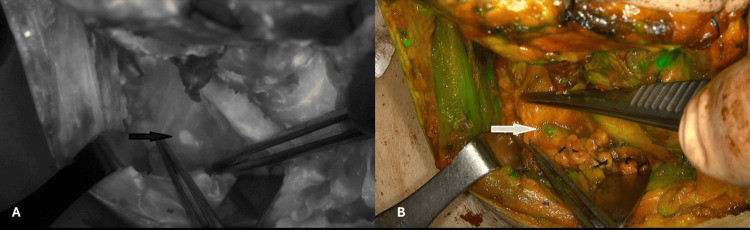
Thoracic duct right side equivalent seen through the right supraclavicular hollow pointed by arrows. (A) Black and white mode, (B) Overlay mode.

In one female patient, 73 years old, with a right-sided papillary thyroid carcinoma metastatic to the right jugular chain and with infiltration of the right internal jugular vein, the equivalent of the thoracic duct was seen on the right supraclavicular hollow with fluorescence in her first surgery (Figure 6).

One female patient, 83 years old, who was operated on eight years before for a large papillary carcinoma without a neck dissection, received only one postoperative dose of I-131 and developed a huge papillary thyroid metastasis on the right side of the neck with airway compression and carotid sheath involvement. She underwent a resection of the metastases that compressed the airway and the carotid sheath. In this case, the equivalent of the thoracic duct on the right side was not visible because of the fibrosis from previous surgery.

In one male patient, 57 years old, with an intrathoracic goiter, the thoracic duct was visible through the left supraclavicular hollow once a retrosternal dominant thyroid nodule was removed. In this patient, approximately 90 minutes elapsed between the injection of ICG in the lymphatic nodes of the inguinal region and the visualization of the TD through the neck.

In none of the three patients mentioned above were the large lymph ducts in the supraclavicular hollow damaged.

**Figure 7:**
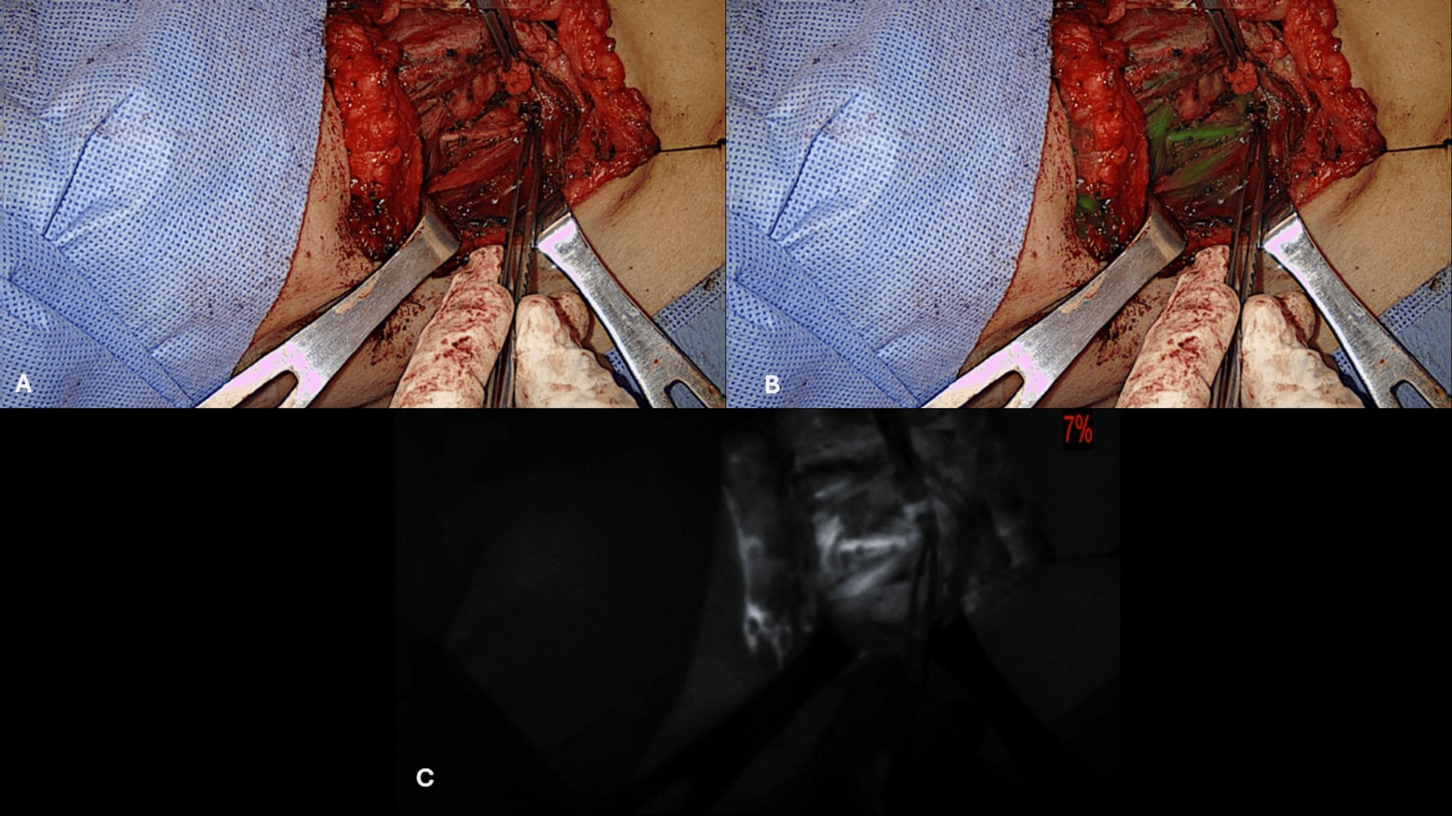
The right recurrent laryngeal nerve. (A) White light mode, (B) Overlay mode, (C) Black and white mode.

**Figure 8:**
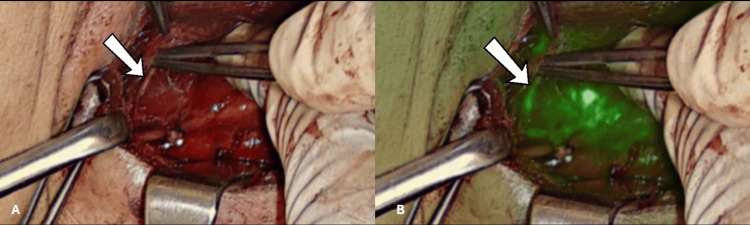
The external branch of the superior laryngeal nerve in (A) white light mode and (B) overlay mode.

During the review of the videos of some of the surgeries, we saw nerves emitting fluorescence in 14 patients. These included recurrent laryngeal nerves either bilaterally or unilaterally (Figure 7) and the external branch of the superior laryngeal nerve (EBSLN) unilaterally in one case (Figure 8).

**Figure 9:**
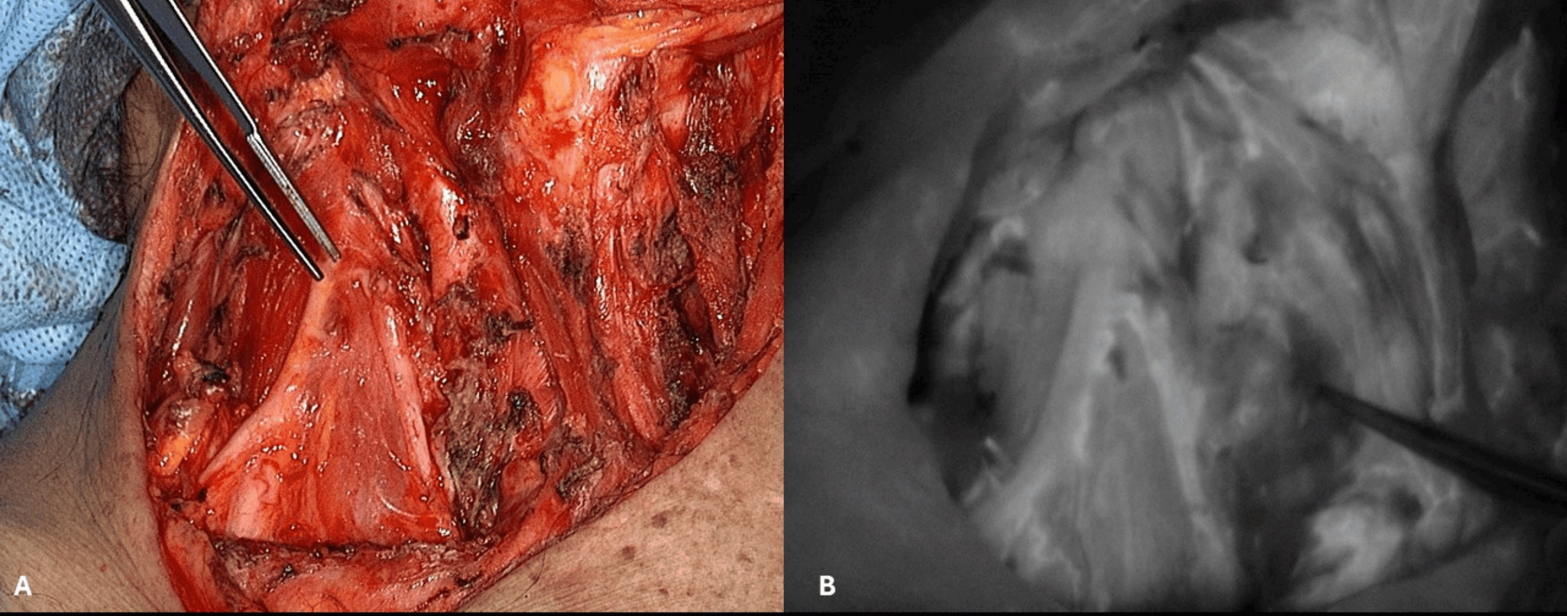
The brachial plexus and the phrenic nerve on the right side. (A) White light mode, (B) Black and white mode.

The main trunk of the right facial nerve with its branches, as seen in Figure 4, and the emergence of the brachial plexus and phrenic nerve on the right side in a patient in which a part of the metastasis had to be left over the common carotid artery because it was firmly attached to the arterial wall (Figure 9). A total of 21 nerves in 12 patients were observed emitting fluorescence. Except for the last three cases, no specific dose for nerve identification was applied; we used the previously administered doses for PG and thoracic duct identification. In one case of combined surgery, left lobe thyroidectomy and right superficial lobe parotidectomy, the remaining deep lobe of the right parotid gland captured ICG after approximately 30 seconds, and the facial nerve did the same after approximately three minutes. The larger the nerve branches were, the longer it took them to emit fluorescence, and the longer they remained with ICG fluorescence emission.

## Discussion

**Figure 10:**
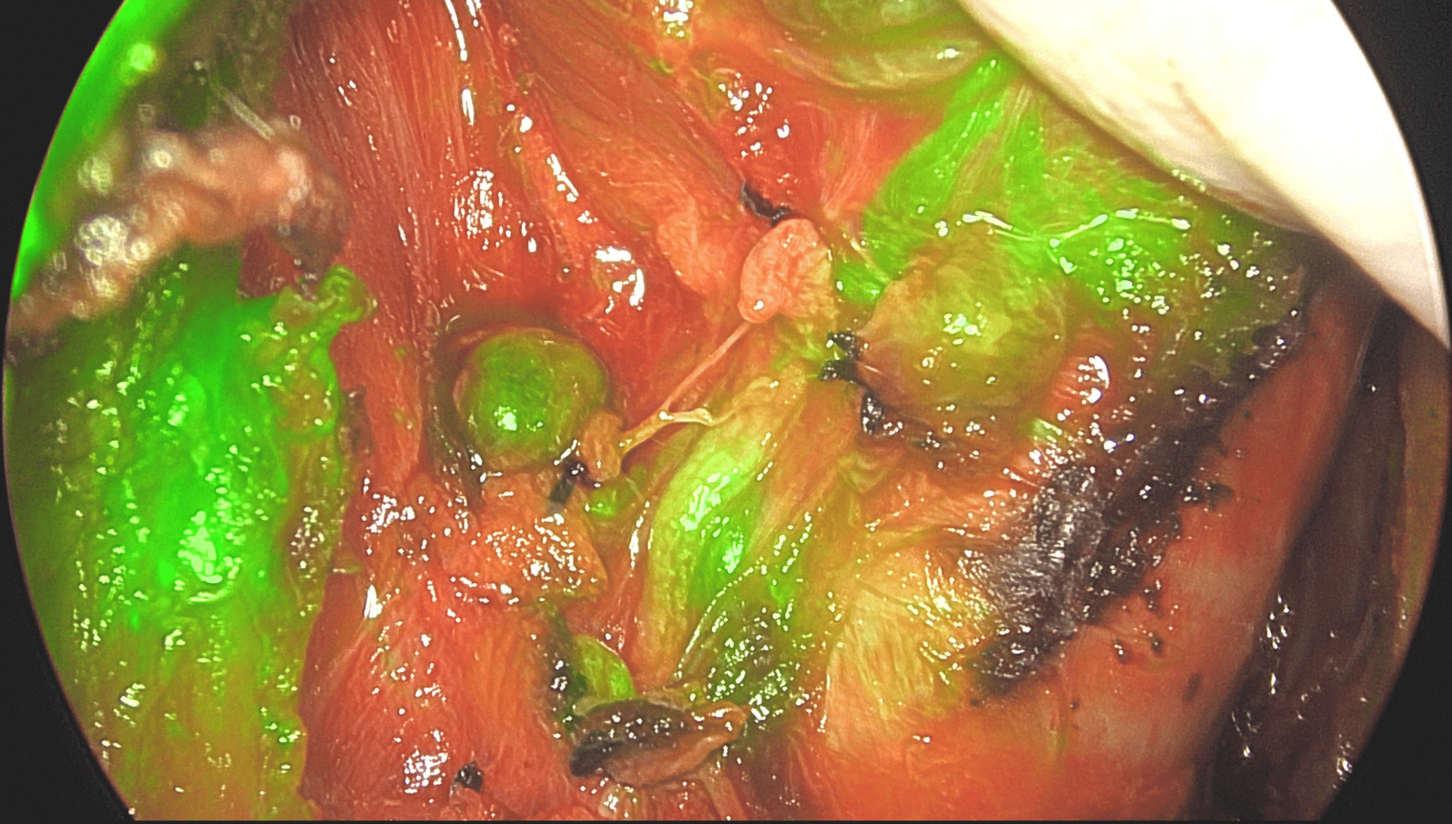
The right superior parathyroid gland emitting fluorescence in overlay mode, the right recurrent laryngeal nerve medial to the parathyroid gland.

FGS with ICG is not a new technique. Reports of its use in ophthalmology and other specialties with different applications date back to 2010 and before [1,5]. Certainly, the most common use nowadays is in liver, biliary tree, gallbladder, and pancreatic surgery. Its utility for colorectal surgery is gaining wide acceptance. The benefit of using ICG for the identification of PGs was first reported by Suh et al. in an animal model [6], after which it was extensively used by different authors [3,4,6,7]. Figure 10 shows the right superior PG and right recurrent laryngeal nerve emitting fluorescence in overlay camera mode in one of the cases in the present study. The purpose of its use is to have an aid to identify and preserve the PGs during thyroid surgery, avoiding accidental resection of the PGs or devascularization, which are important risk factors for the development of PoSH and loss of quality of life [8].

There are other visual techniques for the identification of parathyroid glands during neck surgery, such as carbon nanoparticle (CNP) suspension and laser speckled contrast imaging (LSCI) [9], that were not employed in this study because they are not available to us.

In our literature search of the applications of FGS-ICG for the surgery of the major salivary glands, we could not find an article specific to this matter. A case report of Pasquale et al. describes the resection of a metastasis of an adenoid cystic carcinoma of the parotid gland to the liver, in which FGS-ICG helped to clearly identify the boundaries of the metastasis [10]. One important finding was that although it was a metastatic lesion, the tissue captured avidly the ICG, resulting in complete fluorescent visualization of the metastasis, a feature that is not common in a liver metastasis. Our description of an early and intense capture of ICG by major salivary glands coincides with this finding. Major salivary glands fluorescence with ICG could also be employed during neck dissections for the precise delimitation of the submandibular gland and to distinguish the gland tissue from the lymph nodes, avoiding an unnecessary resection of the gland and resecting only the metastatic lymph nodes in the case that they are found. Embryologically, the only major salivary gland that contains lymphatic nodes inside its parenchyma is the parotid gland. By not resecting the submandibular gland, better salivation could be preserved in older patients POP; in this group of patients, it is common to perform lymph node dissections that include the submandibular glands. From a theoretical point of view, avoiding the resection of the submandibular salivary gland and resecting only the lymph nodes around it could result in a more precise surgery and a better QoL for the patient.

Fluorescence angiography with ICG of the carotid arteries is mentioned in the literature mostly related to retinal diseases, retinal surgery, and cerebrovascular surgery. Greuter et al. used multispectral fluorescence (MFL) imaging with ICG and video angiography that allowed real-time augmented reality visualization of blood flow superimposed on white-light microscopic images during cerebrovascular surgery, and compared it with digital subtraction angiography [11]. Siedek et al. employed intraoperative fluorescence angiography of the carotid artery to confirm the effect and extension of preoperative embolization of two carotid artery paragangliomas (six paragangliomas of the head and neck ) [12]. Apart from the mentioned article, we found no literature reports on the use of fluorescence angiography with ICG of the carotid arteries during the surgical resection of carotid paragangliomas for the delineation of surgical margins and confirmation of complete excision. Carotid paragangliomas in Mexico City are common due to the fact that the average altitude above the sea level of the city is 2300 m, and sometimes patients can present with large (Shamblin III +) masses. We think that carotid artery fluorescence angiography with ICG is a very simple, real-time, and safe resource for this type of surgery.

Apart from using FGS-ICG for fluorescence angiography, we took advantage of the diffusion of the colorant through the lymphatic vessels for the identification of important lymphatic structures in the neck, like the thoracic duct and its equivalent on the right side through the supraclavicular hollow. Identification of the thoracic duct in the thorax with FGS-ICG is well documented during minimally invasive esophagectomy to avoid injury, with consequent chyle leak. There are different techniques that include subcutaneous injection of ICG in the dorsum of the foot, injection of ICG in the inguinal lymph nodes with ultrasound guidance [13], and even in the subcutaneous tissue of the inguinal region [14]; the doses employed are very similar. During our literature search, we found no articles mentioning the attempt to view the thoracic duct, or its equivalent on the right side, through the supraclavicular hollows. We learned that the distance between the external camera and the structure we want to visualize is important; therefore, approaching the camera to the objective must be done carefully. It is important to mention that a chyle leak in the neck is as important as it is in the thorax and requires postoperative drainage, special diet, and sometimes surgical re-exploration. So we consider that the help that FGS-ICG can give to avoid a chyle leak in extensive neck surgery is extremely high. We haven't used FGS-ICG for re-exploration looking for a chyle leak in the neck yet, but it might be a good option for such cases.

Fluorescence of the nerves with ICG has been studied with different protocols and colorants. Kunshan et al. report their trial that includes preclinical and clinical results; they used specially designed video equipment and cameras and large doses of ICG, 5 mg/kg (the highest dose believed to be non-toxic in humans so far), with ICG injection 24 hours preoperatively [15]. Fluorescent images of nerves have been obtained with fluorophores other than ICG [16,17]. FGS-ICG has been employed for the visualization of the facial nerve during parotidectomy and other craniomaxillofacial techniques. Kwon et al. described the use of ICG fluorescence for what they call nervography, based on the detection of fluorescence in the vasa nervorum shortly after the IV injection of ICG [18]. They used a solution of 25 mg of ICG in 5 mL of water, and administered two to three doses without specifying the precise amount of ICG that was administered. They concluded that the use of FGS-ICG helped to preserve facial nerve function in their group of patients. Chen et al. reported the use of FGS-ICG during mastoidectomy for pathology of the cerebellopontine and petroclival regions in 16 patients [19]. They used a solution of 5 cc of pure ICG in 10 mL of water, using 0.2-0.5 mg/kg as the total dose. The visualization of the facial nerve was performed using the infrared mode of an operating microscope. No iatrogenic lesion to the facial nerve was found. Although these studies [18,19] were considered pilot studies by the authors, the benefits of using fluorescence with ICG for the identification of the facial nerve are plausible.

No report of fluorescence ICG visualization of laryngeal nerves during thyroidectomy or of the brachial plexus and phrenic nerve during neck dissection was found in our search of the literature. As per our knowledge, there is no protocol that could be specifically recommended for the detection of fluorescence emission from the nerves with low doses of ICG, as we found. In the last three patients that we reported, a dose of 7.5 mg of ICG was specifically used, and the visualization of laryngeal nerves emitting fluorescence was observed approximately two to three minutes later. The clinical application and practical benefit of the visualization of the described nerves emitting fluorescence in our patients is yet to be determined. We believe that further experimental and clinical work about this subject is warranted.

We are aware of the limitations of this report because of the small number of cases of the different procedures in our series. In PG surgery, the benefit of the application of FGS-ICG has been demonstrated [3]. Nevertheless, the variety of surgical procedures in which we observed fluorescence emission with ICG may stimulate other surgical groups from different surgical specialties to apply the technique, as we consider that FGS is the door to image-guided surgery.

## Conclusions

Application of NIR fluorescence with ICG for surgery of the head and neck was originally conceived for thyroid and parathyroid surgery only, looking to confirm and preserve the PGs and avoid PoSH. As we started to use FGS-ICG in different procedures in the head and neck area, we found some benefits and applications that were rarely or not at all reported before. FGS is an open door to image-guided surgery and precision surgery. The ingenuity of the surgical community shall contribute to the expansion and diversification of its use. 
